# New Activity of a Protein from *Canavalia ensiformis*

**DOI:** 10.3797/scipharm.1404-09

**Published:** 2014-06-16

**Authors:** Vanya Petkova Bogoeva, Lidiya Plamenova Petrova, Anton Aleksandrov Trifonov

**Affiliations:** ^1^ Institute of Molecular Biology „Roumen Tsanev“, Bulgarian Academy of Sciences, “Acad. G. Bonchev“ Str. Bl. 21, 1113, Sofia, Bulgaria.; ^2^ Sofia University, 5, J. Bourchier Blvd., 1164 Sofia, Bulgaria.

**Keywords:** Concanavalin A (Con A), Porphyrin, Anticancer compounds, Fluorescence

## Abstract

Concanavalin A is a legume lectin which preferentially agglutinates transformed cells and shows antitumor effects on human breast carcinoma cells in vitro and in vivo. It is considered as a new potential antineoplastic agent targeting apoptosis, autophagy, and anti-angiogenesis in preclinical or clinical trials for cancer therapeutics, which has recently become the object of intensive study. In the present investigation, we show the capacity of the lectin to bind manganese, gold, iron, and zinc porphyrins: all potential anticancer agents. The interaction of the legume lectin with the studied compounds has been investigated by tryptophan fluorescence, showing conformational changes within the quaternary and tertiary structures of the protein. The binding of Con A with manganese, gold, and iron porphyrins, as well as adenine, was studied by fluorescence quenching. In contrast, the interaction of Con A with zinc porphyrin caused an increase in Trp fluorescence and a red shift of 10 nm of the emission maximum position.

However, the binding of Con A to iron porphyrin was accompanied by a 5 nm blue shift of the emission maximum, and a k_D_ of 0.95 ± 0.13 μM was calculated, respectively. The sigmoidal shape of the curve showed cooperative interactions, which indicated the presence of more than one class of binding site within the Con A molecule for iron porphyrin, confirmed by the Hill slope (h = 1.89±0.46). We have found that the legume lectin interacts with porphyrins and adenine with an affinity (0.14–1.89 µM) similar to that of the non-legume lectin, wheat germ agglutinin. In conclusion, the protein Con A shows new binding activity towards porphyrins with anticancer activities and could find prospective application as a drug delivery molecule that specifically targets cancer cells.

## Introduction

Lectins are widely spread carbohydrate-binding proteins, possessing the ability to agglutinate cells and to precipitate polysaccharides and glycoconjugates. Several of them have been found to be multifunctional, as they possess hydrophobic sites in addition to their carbohydrate-binding ones [1].

Prominent amongst plant lectins is Concanavalin A (Con A), isolated from the seeds of *Canavalia ensiformis* (the Jack bean) [2]. It is the most extensively studied representative of the legume lectin family, also being the first whose primary and three-dimensional structures were resolved [3]. Con A is a homotetrameric protein with a molecular weight of 26.5 kDa per monomer.

It is a mannose/glucose-specific lectin, which, similar to other plant lectins, binds noncarbohydrate ligands such as anilinonaphthalene-sulfonic acid (ANS), toluidinyl-naphthalene-sulfonic acid (TNS), zinc porphyrin, etc. [4–6].

Con A has been drawing scientists’ attention for its remarkable antiproliferative and antitumor activities towards cancer cells. It has been found that the protein has the ability to recognize and destroy tumor cells targeting apoptosis, autophagy, and angiogenesis etc., which reveals new perspectives of its application [7]. Con A preferentially binds transformed cells [8]. It induces apoptosis in human breast carcinoma cells without affecting healthy ones [9].

Interestingly, it was reported that iron (III)-salophene and iron porphyrin exhibited apoptotic and chemotherapeutic effects against cancer cells and an ovarian cancer animal model [10, 11].

This motivated us to investigate the complexes of Con A with porphyrins (Sch. 1), in particular Con A-iron porphyrin, Con A-manganese porphyrin, and Con A-gold porphyrin complexes, as presently there are no published data about these interactions. Additionally, we have found that the protein binds adenine and zinc porphyrin similar to several lectins.

Our results help to elucidate the new binding activity of the protein Con A, aiming to characterize the mechanism of its interactions and affinity constants towards new compounds with established anticancer properties. This may find new perspectives to improve the ability of anticancer agents to target tumor cells specifically.

**Sch. 1.**
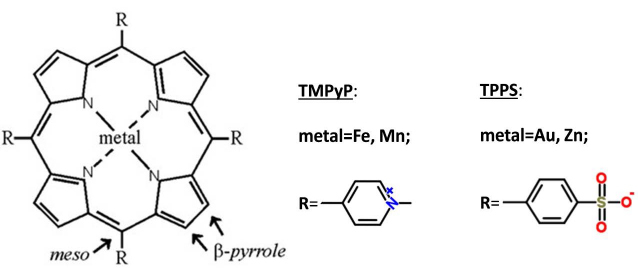
Schematic structure of the studied metalloporphyrins: gold-, iron-, manganese-, and zinc-porphyrins

## Results and Discussion

The hydrophobic binding property of lectins and their possible medical applications have attracted scientific interest for the last several decades. It has also been discussed that carbohydrate binding capacity is not the only activity of plant and animal lectins [12-15]. Discovering the hydrophobic sites of several lectins put forward the question of their novel functions and clinical applications [14].

In this study, we characterized the interaction of the protein Con A with adenine, AuTPPS, MnTMPyP, FeTMPyP, and ZnTPPS using a fluorescence spectroscopy method. We found that excitation at λ=295 nm of Con A shows the Trp emission spectra, which are sensitive to the interactions of the protein with the studied compounds.

**Fig. 1.**
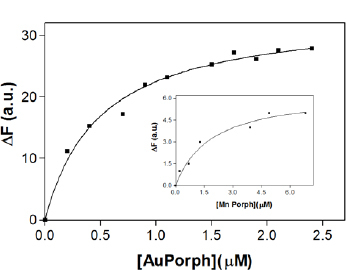
Interaction of Con A with AuTPPS and MnTMPyP (inset). The curves are the best theoretical fits of the analyzed experimental data

The binding of the protein with AuTPPS and MnTMPyP (Fig. 1) and adenine (data not shown) caused a fluorescence quenching of the Trp emission, which is an evidence of conformational rearrangements within the Con A tetramer. The interaction of Con A with porphyrins and adenine caused a small, non-significant shift of the emission maximum upon titration/incubation with the compounds, similar to phycocyanin binding [16].

The KD of (0.49 ± 0.29 μM) for AuTPPS, (1.89 ± 0.54 μM) for MnTMPyP, and (0.38 ± 0.08 μM) for adenine showed high-affinity binding of the Con A for the three ligands.

We have found that Con A interacts with porphyrins and adenine with an affinity similar to that of WGA (wheat germ agglutinin) [1, 17, 18].

**Fig. 2.**
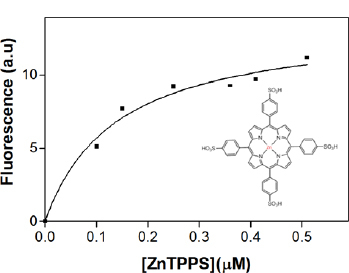
Formation of Con A-ZnTPPS complexes, studied by means of intrinsic protein fluorescence (λ_*exc*_=295 nm) (inset-structure of 5,10,15,20-Tetrakis-(4-sulfonatophenyl)porphyrin-zinc (II))

In contrast, the incubation of Con A with ZnTPPS caused an increase in the Trp fluorescence and a red shift of 10 nm of the emission maximum position was registered. Interestingly, the apparent dissociation constant of the complex was KD=0.14±0.04 μM (Fig. 2), which is almost two orders of magnitude greater than that registered by the porphyrin fluorescence KD=17.7 μM [6].

**Fig. 3.**
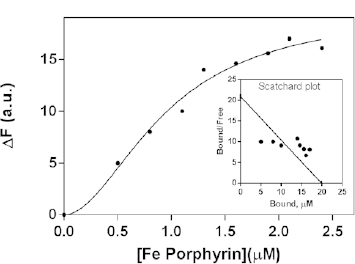
Representative binding curve, showing the formation of Con A-FeTMPyP complexes K_D_=0.95±0.13 μM (inset- Scatchard plot, h=1.89±0.46)

We also registered the interaction of Con A with FeTMPyP, which was accompanied by a 5 nm blue shift to the shorter wavelengths. From the representative curve, the dissociation constant (KD) of 0.95 ± 0.13 μM was calculated using the GraphPad Prism software program. Interestingly, the sigmoid shape of the binding curve (Fig. 3) showed the presence of more than one class of sites for FeTMPyP within Con A.

The curve revealed cooperative interactions which indicated that binding of one FeTMPyP molecule facilitated the interaction of the next FeTMPyP molecules by enhancing the affinity of the vacant binding sites on the lectin. The obtained results demonstrated that the legume lectin Con A bound FeTMPyP with high affinity, similar to that of the non-legume lectin WGA for iron porphyrins [18, 19].

We have calculated that Con A has more than one binding site for FeTMPyP with positive cooperativity, confirmed by the Hill slope (*h*), which is greater than 1.0 *(h =* 1.89 ± 0.46) and by the non-linear distribution of the data in the *Scatchard plot* (Fig. 3 – inset).

Our data are in agreement with the literature data and the general principle describing the protein-ligand interactions. It is established that the binding of a ligand to a protein may directly affect the Trp fluorescence by quenching it or by physical interaction with the fluorophore, thus changing the polarity of the microenvironment and/or accessibility to the solvent.

However, another possibility occurs when the ligand binds at a site distinct from the Trp amino acid and acts by a generalized mechanism, causing the protein conformational changes which alter the Trp microenvironment. Both direct and summarized effects may result either in a fluorescence increase or quenching and/or spectrum shift (blue or red), respectively [20], as it is observed in our case.

Interestingly, it was reported that iron (III)-salophene and iron porphyrin exhibited apoptotic and chemotherapeutic effects against cancer cells in an ovarian cancer animal model [10, 11]. It was also found that MnTMPyP showed a potent synergistic cytotoxic effect when combined with an ascorbic acid in a variety of cancer cell lines (Du-145, HD-LAPC-4, Hep-G2, HI-LAPC-4, LnCAP, PANC-1, and PC-3) [21].

Recently, it has been discussed that gold porphyrins are prospective anticancer compounds against nasopharyngeal cancer cells (NPC) [22]. ZnTPPS is effective in ROS production, which is necessary for photodynamic therapy, and induces cell death in G361 (a human melanoma cell line) [23].

Why is it important to study porphyrins and their interactions with Con A? Since many porphyrin-based drugs cannot accumulate selectively in tumors, the question that obviously arises is whether Con A, considered as a recognition molecule and a novel candidate antineoplastic drug [7], could be applied in drug delivery to cancer cells? The study of Con A-porphyrin complexes is important, since Con A could be viewed as a possible drug-binding molecule with potential application in the drug delivery of cancer.

The property of Con A to interact with metal-based agents with various chemical structures might be due to its unique structure, which consists of four subunits [3]. Besides this, the crystal structural analysis demonstrates a low packing density of the Con A tetramer, which results in protein sidechain rearrangements due to the interaction with the ligands.

As we have discussed before, several groups have studied the hydrophobic binding capacity of Con A using the fluorescent hydrophobic dyes ANS and TNS [4, 5]. It was also found that the porphyrins H_2_TPPS and ZnTPPS bound both Con A saturated with saccharides and the free protein (without sugar) with comparable affinities, suggesting that the interactions could be at a site distinct from the saccharide-binding one [6].

Contrary to this, the crystallographic model showed that H_2_TPPS bound Con A through its sulfonatophenyl group at the monosaccharide binding site. The porphyrin showed multivalency, which resulted in crosslinking similar to that of the agglutination of cells [24].

It has also been reported that Con A, similar to most legume lectins, has binding sites/pockets for hydrophobic ligands [25]. In particular, three characteristic hydrophobic sites of the lectins are found [26]. Interestingly, it has been discussed that Con A binds phycocyanin via two distinct sites, which involves hydrophobic interactions, charged residues, or hydrogen bonding [16].

Regarding these data as well as our results (Tab. 1), we may suggest that the interaction of Con A with four porphyrins and adenine could involve hydrophobic interactions, charged amino acids, or hydrogen bonding.

**Tab. 1.** Dissociation constants (K_D_) of Con A interactions with porphyrins and adenine

Lectin–ligand conjugates	Dissociation constants (K_D_) for the interaction (μM)
	Monitoring intrinsic Con A fluorescence (λ_exc_ = 295 nm and λ_em_ = 338 nm)
Con – AuTPPS	0.49 ± 0.29
Con A – FeTMPyP	0.95 ± 0.13
Con A – MnTMPyP	1.89 ± 0.54
Con A –ZnTPPS	0.14 ± 0.04
Con A – adenine	0.38 ± 0.08

Probably the differences in crystallographic and solution state data are due to the much higher concentrations required for crystallographic study, causing the porphyrin to lose its monomeric form leading to multivalency and crosslinking [14].

In contrast to this, Trp fluorescence is a very sensitive method, registering the intrinsic conformational dynamics of the protein (in solution), suggesting to play a crucial role in porphyrin binding and dissociation.

Interestingly, a putative adenine binding site sandwiched between two Con A dimers was discussed and proposed by Maliarik and Goldstein [27] when adenine binding sites of legume lectins were investigated by photoaffinity labeling.

Current research has focused on the binding activity of Con A to metalloporphyrins in an effort to seek new possibilities to improve the specific drug recognition of cancer cells.

Interestingly, several lectins, including Con A, can recognize cancer cells and the binding of porphyrins to them may increase the selectivity of the complex for cancer cells.

In this regard, the study of Con A-porphyrin complexes could be important from a therapeutic point of view.

In conclusion, Con A shows a new metalloporphyrin binding activity towards potential anticancer agents and could find prospective application as a drug delivery molecule that specifically targets cancer cells.

## Experimental

### Materials

Lectin from *Canavalia ensiformis* (Con A) was purchased from Sigma. The protein was dissolved in PBS (20 mM phosphate buffer containing 0.15 M NaCl, pH 6.8) and the concentration of Con A stock solution was determined spectrophotometrically, using the extinction coefficient calculated from the aromatic amino acid residues (7 Tyr and 4 Trp: 40, 88, 109, 182 [28] per monomer; [3]). 5,10,15,20-Tetrakis(4-sulfonatophenyl)-porphyrin-Au(III)chloride (AuTPPS), 5,10,15,20-Tetrakis (4-sulfonatophenyl)-porphyrin-zinc(II) (ZnTPPS), 5,10,15,20-Tetrakis (N-methyl-4-pyridyl)-porphyrin-Mn(III)pentachloride (MnTMPyP), and 5,10,15,20-Tetrakis (N-methyl 4-pyridyl)-porphyrin Fe(III) (FeTMPyP) were purchased from Porphyrin Systems (Lüebeck, Germany). ZnTPPS and MnTMPyP concentrations were calculated by their absorbances at 422 nm and 462 nm, respectively, ε_422_ = 3.7 × 10^5^ M^-1^ cm^-1^ for Zn-porphyrin and ε_462_ nm = 0.95 × 10^5^ M^-1^ cm^-1^ for Mn-porphyrin [29, 30]. The concentration of Au-porphyrin was determined at 405 nm (ε_405_ nm = 2.82 - 10^5^ M^-1^ cm^-1^) [30] and adenine concentration was also determined spectrophotometrically, using the extinction coefficient at 260 nm (ε_260_ = 13 × 10 M^-1^ cm^-1^) [31].

### Fluorescence Measurements

Fluorescence spectra were recorded with a Shimadzu spectrofluorometer (Japan). The emission spectra of the tryptophan residues were recorded in a 1-cm quartz cell (Hellma Analytics, Germany). To avoid the measurement of the Tyr emission, the protein sample was excited at λ=295 nm with a slit width of 1.0 nm. Total fluorescence was calculated after the normalization of the fluorescence spectra and correction for dilution. In order to normalize the inner filter and self-absorption effects, the excitation was always carried out less than 0.05 OD. The spectra were recorded in a wavelength range of 300–500 nm. All studies were done at 25°C with the temperature of the samples measured in the cuvette with an accuracy of ± 0.2°C.

### Analysis of the Interaction of Con A with Porphyrins

The protein-ligand interactions were investigated by monitoring changes in the intrinsic protein fluorescence emission (θ_ex_c=295 nm). We applied ideal fluorescence titration and incubation methods for their intrinsic sensitivity and simplicity. For this purpose, Con A (1.8 µM) was incubated at 4°C overnight with increasing concentrations of MnTMPyP, ZnTPPS, and FeTMPyP. Also, Con A (1.5 μM) was titrated with increasing concentrations of AuTPPS. Applied porphyrin concentrations were consistent with minimizing artifacts due to aggregation phenomena. Con A-adenine interactions were studied by overnight incubation (at 4°C). The obtained experimental data were processed by non-linear and linear regression analysis, computed by a GraphPad Prism program and presented in figures comprising three independent sets of experiments.
